# 1026. Hepatitis B Reactivation in Persons with HIV with Positive Hepatitis B Core Antibody after Switching to Antiretroviral Therapy without Hepatitis B Activity

**DOI:** 10.1093/ofid/ofad500.057

**Published:** 2023-11-27

**Authors:** Rachel V Denyer, Janet P Tate, Debra A Benator, Joseph K Lim, Amy Weintrob

**Affiliations:** Washington DC VA Medical Center/ George Washington University, Washington, DC; VA Connecticut Healthcare System/ Yale University School of Medicine, West Haven, Connecticut; Washington DC VA Medical Center, Washington DC, DC; Yale University School of Medicine, New Haven, Connecticut; Washington DC VA Medical Center/ George Washington University, Washington, DC

## Abstract

**Background:**

One in three persons with HIV (PWH) without active hepatitis B virus (HBV) coinfection have a positive hepatitis B core antibody (cAb) suggesting prior HBV exposure. This group is at risk for HBV reactivation (HBVr) when changes to antiretrovirals (ARV) result in cessation of nucleos(t)ide reverse transcriptase inhibitors (NRTIs) with dual activity against HIV and HBV, e.g. tenofovir. Case reports of HBVr in PWH with positive cAb after NRTI cessation have been published, but the frequency of reactivation is unknown. Since FDA approval of dolutegravir-rilpivirine in 2017 and long-acting injectable cabotegravir-rilpivirine in 2021, increasing numbers of PWH are switching to ARV without HBV activity. We describe HBV reactivation in cAb positive PWH following switch to ARV without HBV activity (AHB-) from ARV with HBV activity (AHB+).

**Methods:**

We used data from 60,290 PWH enrolled in the Veterans Aging Cohort Study to identify 7,860 who switched to AHB- prior to 12/31/2022 with prior positive cAb and negative pre-switch hepatitis B surface Ag (sAg). We excluded 779 due to active HBV (positive sAg or hepatitis B DNA [HBDNA] on nearest result pre-switch), leaving 7,081 eligible for analysis. We included PWH with remote prior positive sAg not meeting active HBV criteria. We defined HBVr as detection of HBDNA or sAg. AHB+ included lamivudine, emtricitabine, and/or tenofovir.

**Results:**

HBVr occurred in 1.6% of PWH with positive cAb (n = 115) after switch to AHB-, with demographics summarized in Table 1. Table 2 shows prespecified subgroup analyses by prior sAg and hepatitis B surface antibody (sAb). Reactivation risk was 1.0% in those with no prior positive sAg and 20.2% in those with remote prior positive sAg (p < 0.0001). Among those with no prior positive sAg, HBVr risk in those with positive sAb was 0.4%, compared with 1.1% in those without positive sAb (p = 0.065); among those with remote prior positive sAg, no difference in risk by sAb status was found (p = 0.64).

**Figure:**
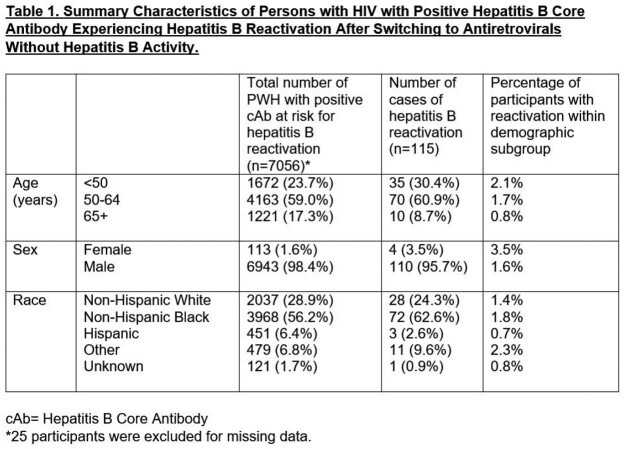

**Figure:**
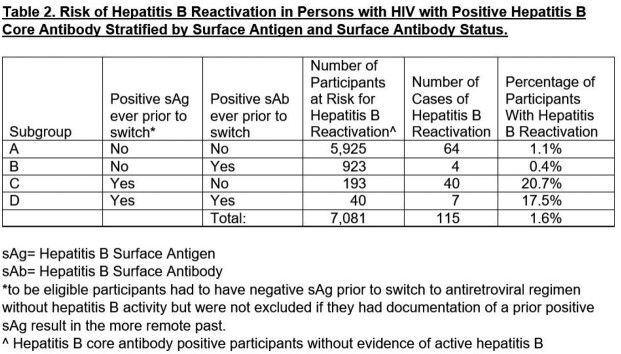

**Conclusion:**

Overall risk of HBVr is low after AHB+ to AHB- switch among cAb positive PWH with no prior positive sAg. Our results will inform provider-patient discussion about risks of HBVr when considering switching to newer NRTI-sparing AHB- and also highlight the importance of pre-switch review of all prior hepatitis serology results.

**Disclosures:**

**All Authors**: No reported disclosures

